# Dataset of Windows operating system forensics artefacts

**DOI:** 10.1016/j.dib.2024.110693

**Published:** 2024-06-28

**Authors:** Eva Marková, Pavol Sokol, Sophia Petra Krišáková, Kristína Kováčová

**Affiliations:** Pavol Jozef Šafárik University in Košice, Faculty of Science, Institute of Computer Science, Slovakia

**Keywords:** Digital evidence analysis, Event logs, Filesystem, Forensic artefact, NTFS, Operating system Windows

## Abstract

The dataset consists of records from the NTFS file system and event logs. In this study, we used images of devices from capture the flags competitions focused on the digital forensic of Windows operating systems and user activities. We created timelines of the security incident from the disk images using the Plaso tool, which we then processed and transformed the attributes of the timelines into binary values to simplify the application of data analysis and machine learning methods. The data are divided into 12 different files, and they are saved in CSV format.

Specifications Table

**Table:** 

Subject	Computer Science
Specific subject area	Cybersecurity, digital forensics.
Type of data	Tabular data (main CVS files) containing data from event logs and NTFS file system from the Windows operating system.
Data collection	The images of devices (computers, laptops) were downloaded from various CTF competitions websites and storages. The goal of this CTFs is to provide experience related to the field of digital forensics, incident response and threat hunting. We have used the data from the case titled The Stolen Szechuan Sauce from the DFIR Madness Portal, data from Magnet CTFs (2019, 2020, 2022) and data from NIST data leakage case and data hacking case. The data are freely available at the portal and are used for training purposes regarding incident response, and digital forensics for beginners but also professionals in the field.
Data source location	The analyzed and pre-processed data are disk image files from the domain controller and also disk image files from the desktop from the case titled The Stolen Szechuan Sauce. Specifically, we have used files DC01 Disk Image (E01) and Desktop Disk Image (E01). Next image files are Magnet CTF 2019 Windows Desktop, Magnet CTF 2020 Windows Desktop, Magnet CTF 2022 Windows Laptop, and NIST Data Leakage Case.
Data accessibility	Repository name: Mendeley Data - Dataset of Windows operating system forensics artefacts [1] Data identification number: https://dx.doi.org/10.17632/8dfh724hvc.1 Direct URL to data: https://data.mendeley.com/datasets/8dfh724hvc/1
Related research article	P. Sokol, Ľ. Antoni, O. Krídlo, E. Marková, K. Kováčová, S. Krajči, Formal concept analysis approach to understand digital evidence relationships, International Journal of Approximate Reasoning. 159 (2023). https://doi.org/10.1016/j.ijar.2023.108940

## 1 Value of the Data

• The data enables the use of data analysis and machine learning in digital forensic analysis. NTFS is the most widely used file system within the Windows OS. For this reason, these data represent a key example of the application of data analysis and machine learning methods in the field of cyber security, specifically in digital forensic analysis. For example, in our research, we have applied formal concept analysis [2,3] or outlier detection methods [4].
• These data are suitable for researchers dealing with the application of data analysis and machine learning in cybersecurity, but also for professionals who want to automate the process of incident response and digital forensic analysis.
• The data can be used for the development of possible automated tools to identify relevant digital evidence, relationships between evidence, their attributes, and others.
• Identifying digital evidence and behavioral patterns can help create better tools to detect and prevent cyberattacks. Organizations could more effectively protect their information and data from security threats.
• The presented dataset can be also used in the field of predictive analysis and prevention of security threats. Applying data analysis and machine learning to this data can provide opportunities to identify patterns and behaviors that indicate potential security risks or anomalies in the IT infrastructure.

## 2 Background

An important aspect of digital forensics data research is the ability to create a dataset that meets certain expectations and requirements. In general, there is no dataset that can be used for all research purposes in the field of digital forensics [5,6]. There are several challenges researchers face when using, creating, and sharing datasets. It is important to note that, in general, such datasets are missing in this area, or there is a lack of documentation and formal description of its construction [7,8]. For the purposes of our research, we need to work with a dataset that describes real scenarios that can occur in real security incidents. The goal is to create a suitable dataset for comparing methods in the analysis of digital evidence, which could be used to investigate various problems. In general, there are various datasets that researchers work with, but either they do not share them or not all of them are usable in this type of research. There are also frameworks that can be used to generate datasets, but there is no guarantee that the dataset is correctly created and meets all the conditions necessary for this type of research.

## 3 Data Description

The main goal of creation of a dataset in the area of digital forensics and incident response is to help to automate identification of relevant digital evidence, finding a starting point from which a forensic analyst can start an investigation, and identification relationships between digital evidence and their attributes. This data can significantly contribute to the automation of the process of solving security incidents and digital forensic analysis and can also be used to create automated tools for identifying patterns of behavior in the systems. We decided to prepare tabular data from disk images because for machine learning methods it is necessary to have tabular data as input, while standard acquired digital evidence, such as a forensic disk image, are not suitable for automating digital forensic analysis. The research questions for which this dataset can be used can be diverse and include a wide range of aspects of digital forensics and incident response.

Firstly, we have created supertimelines generated with the plaso tool [9] for all disk images and we have generated 12 auxiliary files with preprocessed data. The preprocessing phase consisted mainly of binarization of the attributes. We decided to use the disk images from Capture The Flag (CTF) competitions focused on the digital forensics, incident response and threat hunting.

There are some limitations while using CTF competitions. CTFs often represent artificially created situations, focused on a specific type of attack carried out by one attacker, which might not reflect the reality where there can be multiple attackers at the same time. Moreover, it is always expected to find something in the CTF data, which may not be true in the case of real security incidents, where we assume uncertainty and complexity. However, some features and characteristics of CTF competitions may also have parallels with real scenarios of security incidents. For example, although CTF tasks often simplify situations and focus on specific types of attacks, much like in the real world, attackers may use specialized tools and tactics to achieve their goals. In addition, although in CTF competitions there is always an expectation to find something that may be obvious and the solution is often structured in a question/answer format, in real incidents this can contribute to faster discovery and resolution of the problem. Another similarity may be the examination of data and digital evidence, which is equally important in CTF competitions and in real digital forensics. Although evidence may be artificially created in CTF tasks, the process of data analysis and extraction may be similar to that of real security incidents.

We used CTF competitions as a source for creation of dataset for digital forensics because access to real data from security incidents can be problematic and limited. Data from real-life security incidents is often not publicly available and obtaining it can be difficult due to permissions, legal restrictions, or privacy protections. In this paper, we focus on Windows disk images from CTFs because the field of digital forensics is very broad. Each of the subfields, such as network forensics, Windows, Linux, or mobile forensics, is specific and requires different preprocessing and analysis. Data is structured differently in each of these areas and needs to be accessed using different methods.

In our dataset we have preprocessed the following cases:

**The Stolen Szechuan Sauce** [10]

The main aim is to determine how a secret Szechuan sauce recipe belonging to CITADEL company ended up on a dark website. The company requested a forensic analysis of its Domain Controller and network host to identify malicious applications installed on the system and determine place and time of software installation. The case also provides us with information – whether any information has been created, modified or deleted and whether there has been a data breach. We are working with artefacts from the company's Domain controller server (DC server) and from the Desktop (network host).

**Magnet / CTF 2019** [11], **Magnet / CTF 2020** [12], and **Magnet / CTF 2022** [13]

All of the three disk images were part of the capture the flag competition. It is not a classic process of digital forensic analysis, but rather answers to specific questions, such as “when was the disk image acquired,” “when was the software installed,” and others.

**NIST / Data Leakage Case** [14]

The purpose of this disk image is to learn various types of data leakage, and practice its investigation techniques. This is an instance of data breach investigation where we need to uncover evidence of the wrongdoing and any information potentially created by the suspect.

The first step was creation of supertimelines for all images using plaso tool with l2tcsv output format. Supertimelines consist of 17 fields - date, time, timezone, MACB, source, sourcetype, type, user, host, short, desc, version, filename, inode, notes, format and extra.

Modified event logs

The files (<case>_evt.csv) are created from the timelines. The original attributes were modified and also new attributes were extracted. We provide the description of the attributes:

1. **Column1** – the original order of record in the file containing the timeline
2. **inode** – the original inode column, the designation of the Inode within the NTFS file system
3. **id** – uniquely generated unique id
4. **datetime** – time stamp, joined date and time columns, UTC time zone
5. **computer_name** – extracted from desc column, or column extra
6. **source_name** – extracted from desc column, or column extra - log source (e.g. Service Control Manager)
7. **filename_type**
8. **filename_security** – extracted from the filename, log column within Security.evtx
9. **filename_application** – extracted from the filename, log column within Application.evtx
10. **filename_system** – extracted from the filename, log column within System.evtx
11. **filename_rdp** – extracted from the filename column, log under Microsoft-Windows-RemoteDesktopServices-RdpCoreTS%4Operational.evtx
12. **filename_powershell** – extracted from the filename column, log within Microsoft-Windows-PowerShell%4Operational.evtx
13. **filename_other** – extracted from the filename column, the log did not appear in any of the aforementioned groups at filename
14. **recovered** – extracted from the extra column, True if the record was recovered (recovered data = deleted data)
15. **user_sid** – extracted from the extra column, the ID of the user who created the process
16. **execution_process_id** – extracted from the extra column, the ID of the process being executed
17. **channel** – extracted from the extra column, e.g. System
18. **event_id** – a specific type of event
19. **audit_account_logon** – divided on the basis of evt groups - into 9 groups, determined by Event ID
20. **audit_account_management** – divided on the basis of evt groups - into 9 groups, determined by Event ID
21. **audit_detailed_tracking** – divided on the basis of evt groups - into 9 groups, determined by Event ID
22. **audit_logon_logoff** – divided on the basis of evt groups - into 9 groups, determined by Event ID
23. **audit_ds_access** – divided on the basis of evt groups - into 9 groups, determined by Event ID
24. **audit_object_access** – divided on the basis of evt groups - into 9 groups, determined by Event ID
25. **audit_policy_change** – divided on the basis of evt groups - into 9 groups, determined by Event ID
26. **audit_privilege_usage** – divided on the basis of evt groups - into 9 groups, determined by Event ID
27. **audit_system** – divided on the basis of evt groups - into 9 groups, determined by Event ID
28. **task** – extracted from the extra column. Gets a task identifier for a portion of an application or a component that publishes an event. A task is a 16-bit value with 16 top values reserved. This type allows any value between 0×0000 and 0xffef to be used.
29. **keywords**
30. **keywords_audit_failure** – AuditFailure = 0×10000000000000L, Attached to all failed security audit events. Use this keyword only for events in the security log.
31. **keywords_audit_success** – AuditSuccess = 0×20000000000000L, Attached to all successful security audit events. Use this keyword only for events in the security log.
32. **keywords_correlation_hint** – CorrelationHint = 0×10000000000000L, Attached to all successful security audit events. Use this keyword only for events in the security log.
33. **keywords_event_log_classic** – EventLogClassic = 0×80000000000000L, Attached to events that are raised by using the RaiseEvent function.
34. **keywords_sqm** – Sqm = 0×08000000000000L, Attached to all Service Quality Mechanism (SQM) events.
35. **keywords_wdi_context** – WdiContext = 0×02000000000000L, Attached to all Windows Diagnostics Infrastructure (WDI) context events.
36. **keywords_wdi_diagnostic** – WdiDiagnostic = 0×04000000000000L, Attached to all Windows Diagnostics Infrastructure (WDI) diagnostic events.
37. **timestamp** – modified timestamp (date and time)
38. **epochtime** – epochtime (from 1.1.1970)
39. **hour** – hour (extraction from timestamp)
40. **minute** – minute (extraction from timestamp)

In Table 1 we present the basic quantitative data that describe the evt datasets, including the size of the dataset in kilobytes, the number of records, the number of unique inodes, SIDs, computer names and event IDs. These data provide an overview of the structure and scope of the dataset, which is crucial for its analysis and use in various research contexts.

**Table 1:** Information about evt datasets.

	The Stolen Szechuan Sauce Case DC	The Stolen Szechuan Sauce Case Desktop	Magnet CTF 2019 Windows Desktop	Magnet CTF 2020 Windows Desktop	Magnet CTF 2022 Windows Laptop	NIST Data Leakage Case
Size of dataset [kB]	21 510	12 208	31 208	34 669	69 835	2 689
Number of records	86 180	41 721	111 102	130 359	251 666	10 306
Number of unique inodes	81	113	116	50	232	34
Number of unique SIDs	14	18	18	6	16	8
Number of unique computer names	4	3	2	2	2	74
Number of unique event IDs	464	644	612	289	701	211

Modified file system events

The files (<case>_file.csv) are created from the timelines. The original attributes were modified and also new attributes were extracted. We provide the description of the attributes:

1. **Column1** – the original order of record in the file containing the timeline
2. **filename** – file name
3. **inode** – the original inode column, the designation of the Inode within the NTFS file system
4. **extra** – column with information - from this column were extracted different attributes
5. **id** – a column with a generated unique id (for record retrieval)
6. **datetime** - time stamp, joined date and time columns, UTC time zone
7. **M** – modified
8. **A** – accessed
9. **C** – changed, $MFT modified
10. **B** – birth, file creation time
11. **file_stat** – a column based on values from the sourcetype column
12. **NTFS_file_stat** – a column created based on the values from the sourcetype column
13. **file_entry_shell_item** – a column based on the values from the sourcetype column
14. **NTFS_USN_change** – a column based on the values from the sourcetype column
15. **file_path** – file with the full path
16. **name** – column name extracted from desc
17. **typef** – file type ('None','file','directory','link')
18. **filef** – 1 if it is a file, 0 if not
19. **directory** – 1 if it is a folder, 0 if not
20. **link** – 1 if it is a link file, 0 if not
21. **dir_type** – directory type - file location ('Windows', 'AppData','Other','Users')
22. **dir_appdata** – path description - extracted column - based on values from desc, 1 if AppData is in the path
23. **dir_win** – path description - extracted column - based on values from desc, 1 if Windows is in the path
24. **dir_user** – path description - extracted column - based on values from desc, 1 if User is in the path
25. **dir_other** – path description - extracted column - based on values from desc, 1 if none of the three above
26. **file_type** – file type by extension
27. **file_executable** – file type by extension - column extracted based on file type values from filename (executable files)
28. **file_graphic** – file type by extension - column extracted based on file type values from filename (graphic files)
29. **file_documents** – file type by extension - column extracted based on file type values from filename (document files)
30. **file_ps** – file type by extension - column extracted based on file type values from filename (powershell files)
31. **file_other** – file type by extension - column extracted based on file type values from filename (rest)
32. **mft** – a column created based on the values of the format column
33. **lnk_shell_items** – a column created based on the values of the format column
34. **olecf_olecf_automatic_destinations/lnk/shell_items** – a column created based on the values of the format column
35. **winreg_bagmru/shell_items** – a column created based on the values of the format column
36. **usnjrnl** – a column created based on the values of the format column
37. **is_allocated** - column from the extracted value from the extra column (1 if information is found)
38. **is_allocated0** – 1 if the value in the is_allocated column was one (the file is located in the file system)
39. **is_allocated1** – 1 if the value in the is_allocated column was zero (the file was deleted)
40. **file_size** – file size
41. **sha_256** – the hash extracted from the extra column
42. **timestamp** – modified timestamp (date and time)
43. **epochtime** – epoch time (from 1.1.1970)
44. **hour** – hour (extraction from timestamp)
45. **minute** – minute (extraction from timestamp)

In Table 2, we present information about the file datasets, which include the size in kilobytes, the number of records, the number of unique inodes and filenames. These data provide a detailed overview of the structure and scope of individual file datasets.

**Table 2:** Information about file datasets.

	The Stolen Szechuan Sauce Case DC	The Stolen Szechuan Sauce Case Desktop	Magnet CTF 2019 Windows Desktop	Magnet CTF 2020 Windows Desktop	Magnet CTF 2022 Windows Laptop	NIST Data Leakage Case
Size of dataset [kB]	348 965	350 291	760 543	2 527 120	4 360 616	764 325
Number of records	843 863	881 454	1 862 397	6 655 042	10 669 920	1 907 945
Number of unique inodes	85 975	100 691	143 644	188 396	331 640	77 870
Number of unique filenames	111 964	129 315	194 370	616 514	1 111 412	196 479

**Fig. 1:**
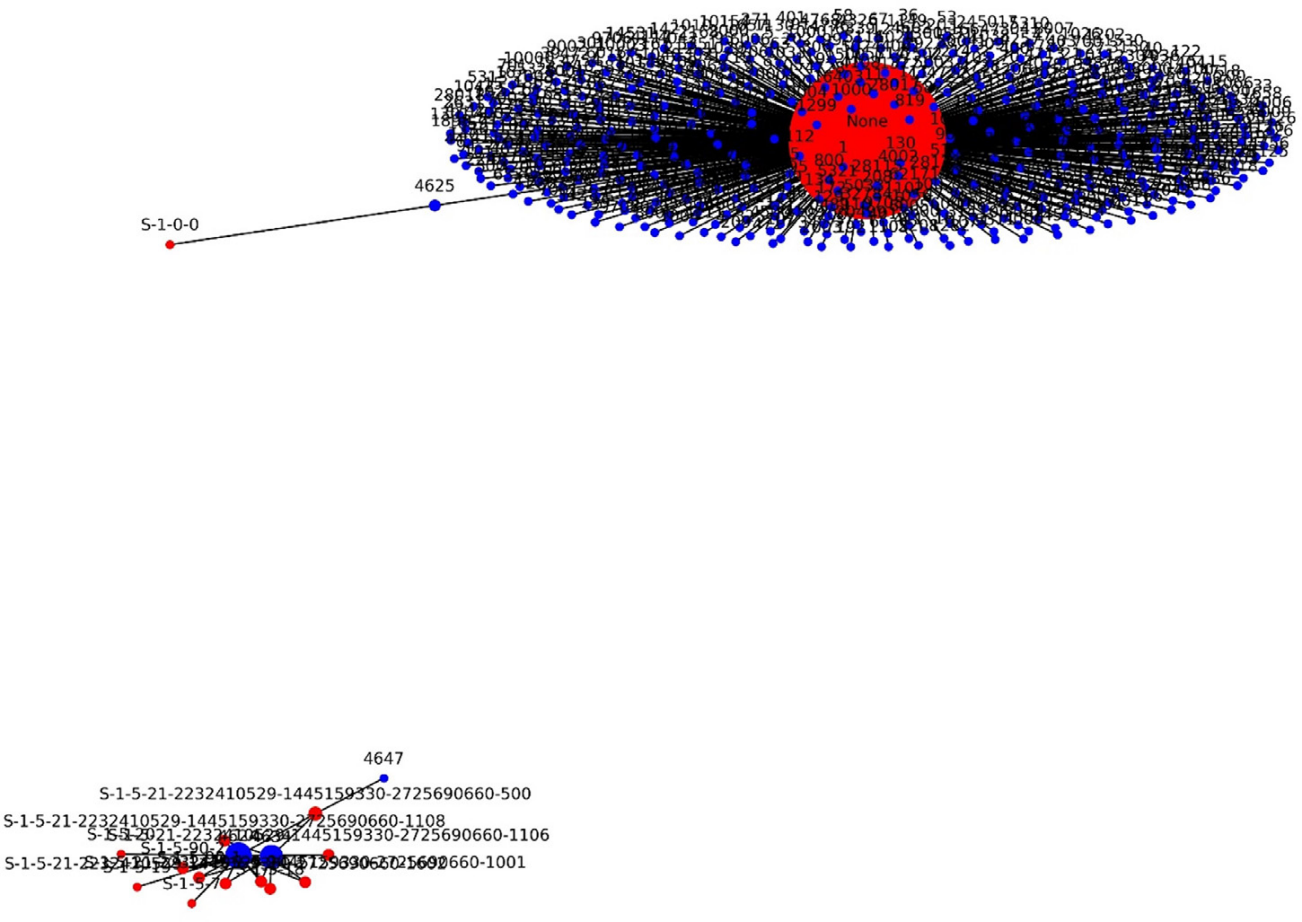
Relationships between the attributes event_id and user_sid.

As an example output using mentioned files we provide the figures. The Fig. 1 contains graph representing the relationship between the attributes event_id and user_sid in the Stolen Szechuan Sauce dataset DC EVT. User_sid attributes are depicted by red nodes, while event_id attributes are represented by blue nodes. If a dataset contains a value for event_id, and in the same row, there is also a value for user_sid, we connect these two values by adding an edge between the corresponding nodes. In other words, if the value of a blue node (event_id) and the value of a red node (user_sid) coexist in the same row of the dataset, we add an edge between these two nodes, thus creating the depicted graph. In this manner, we can generate graphs for various attributes in different datasets (EVT, FILE) and explore relationships between individual attributes. Initially, this exploration is visual, leveraging the graphical expressiveness of the generated graphs. In the second step, we can apply various graph algorithms to analyze cycles, shortest paths, anomalies, and other patterns within the graph. In this figure we can see two separate graphs. In digital forensics point of view, we could analyse for example why there is only one edge from SID „S-1-0-0“, or why two independent graphs are created.

**Fig. 2:**
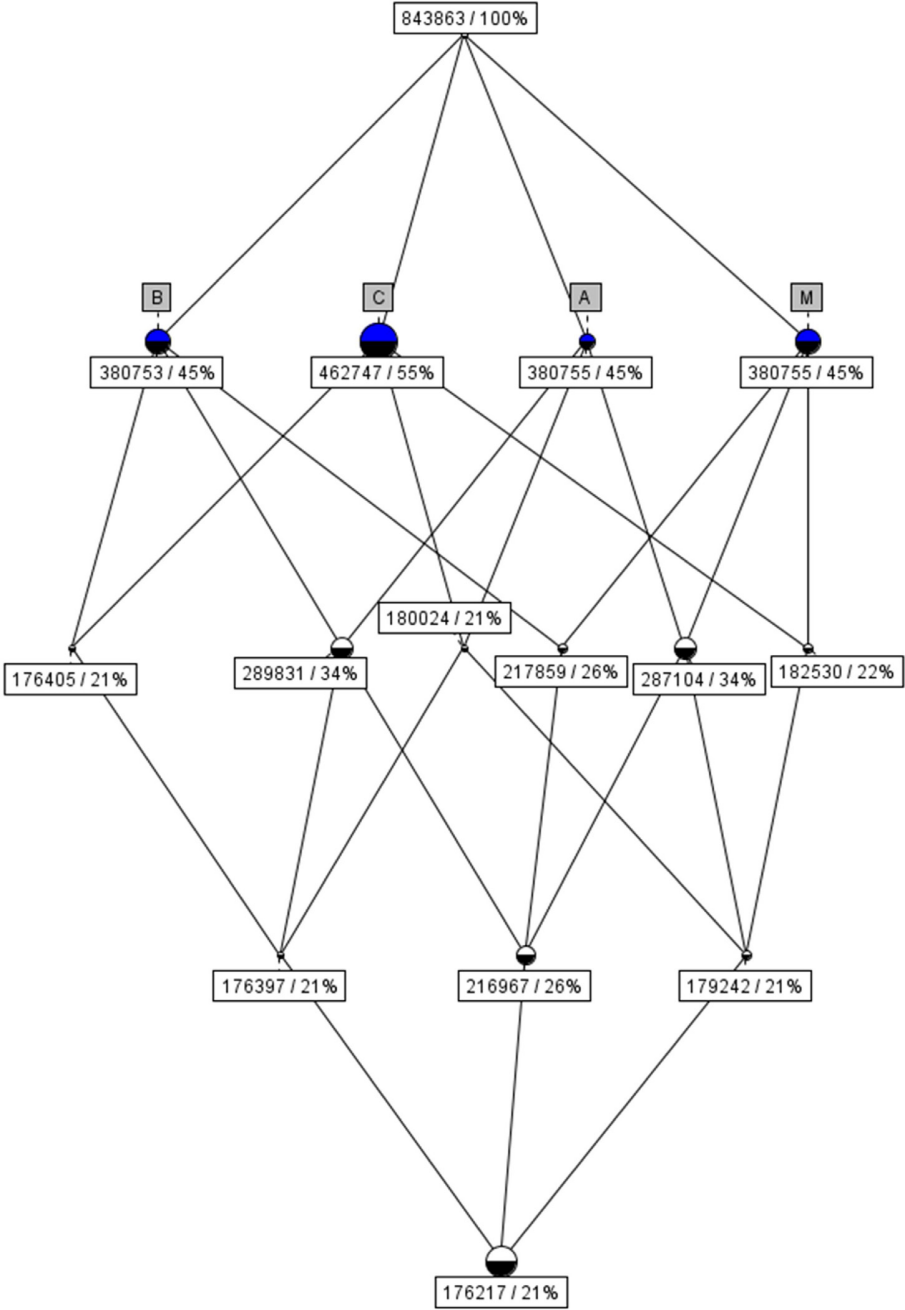
MACB concept lattice.

In our research [2,3] we have used the Formal Concept Analysis on this dataset. In Fig. 2 is the MACB concept lattice shown with 15 vertices representing formal concepts and 28 edges. The height of the concept lattice is 4. At each peak, the number of objects that are part of the concept is indicated, as well as their percentage expression. For example we can see, that 45% (380 753) records contain timestamp B (it means, that B timestamp is presented in that amount of records). Up to 21% (176 217) of all records contain all timestamps (M, A, C, B). The presence of all timestamps represents creation of file.

Association rules take statistical relevance into account. In Table 3, we present the rules for MACB attributes. The expected behavior group emphasizes operating system standards. Records with 90–100% confidence are interesting for digital forensics, especially when identifying suspicious cases in the NTFS file system. It begs the question for a security analyst why this rule doesn't apply in 0.01% of the cases.

**Table 3:** Association rules for MACB attributes.

Association rule	Confidence	Support	Behaviour
{M, C, B} => {A}	100%	20.88%	Expected
{C, B} => {A}	99.99%	20.90%	Suspicious
{A, C, B} => {M}	99.90%	20.90%	Suspicious
...	...	...	...
{M} => {A}	75.40%	45.12%	
{} => {C}	54.84%	100%	

The methods like formal concept analysis or graph theory can be used to find relationships between the digital evidence and their attributes. The anomaly or outlier detection methods might be used to find the relevant digital evidence or to find starting point where forensic analysts should start investigating the security incident.

When collecting and processing any data, it is essential to consider legal and ethical aspects. The first issue to be examined is data privacy and personal data protection. Real data from security incidents are highly valuable, but their biggest issue is precisely this question. This problem is addressed by datasets from Capture The Flag (CTF) competitions. These data are generated by simulating the steps of the attackers, and thus do not contain real data, including sensitive information. Additionally, randomly generated files are used. For example, in the case of the NIST / Data Leakage Case [14], any evidence of data leakage, and any data that might have been generated from the suspect's electronic devices [15]. In the case of data from CTF competitions, there is no breach of data privacy and personal data protection.

The second legal issue that needs to be addressed concerning datasets is copyright. The images provided for the CTF challenges are typically meant for educational and research purposes. In our research, we processed data from five sources from CTF competitions. All these datasets are publicly available, and we provide citations for these sources in this paper. The dataset we have published is derived from these works but constitutes a new, independent work.

Magnet / CTF 2019 [11], Magnet / CTF 2020 [12], and Magnet / CTF 2022 [13] were obtained from the Digital Corpora platform. It is a website of digital corpora for use in computer forensics education and research. All of the disk images, memory dumps, and network packet captures available on this website are freely available and may be used without prior authorization or IRB approval [16]. In the case of The Stolen Szechuan Sauce [10], we have the author's permission for research purposes. Finally, in the case of NIST / Data Leakage Case [14], the data were obtained from the NIST Computer Forensic Reference Data Sets (CFReDS) portal. Investigators could use CFReDS in several ways, including validating the software tools used in their investigations, equipment check-out, training investigators, and proficiency testing of investigators as part of laboratory accreditation [17]. We believe this implicitly includes research purposes.

The third legal issue is the possibility of data misuse. In this case, the data are created for CTF competition purposes. They do not represent any organization or individual, and thus there is no risk of misuse against specific subjects. Potential images may contain malicious code that could be exploited. The datasets we created extract attributes from these images and contain only text data.

## 4 Experimental Design, Materials and Methods

Cases and creation of timelines

As we mentioned, we have analysed five specific cases, six datasets:

1. The Stolen Szechuan Sauce Case– Domain controller (DC)
2. The Stolen Szechuan Sauce Case– Desktop
3. Magnet CTF 2019 Windows Desktop
4. Magnet CTF 2020 Windows Desktop
5. Magnet CTF 2022 Windows Laptop
6. NIST Data Leakage Case

For creation of the timelines of data, we applied the log2timeline tool to the disk images. This tool has many parsers, of which we used win7 slow due to the operating systems, which includes three other parsers, namely win gen, webhist, and win7. The result were files in plaso format, which we subsequently converted using the parser (psort.py) to the l2tcsv format. This format is a simple CVS file with 17 default fields (Date, Time, Timezone, MACB, Source, Sourcetype, Type, User, Host, Short, Desc, Version, Filename, Inode, Notes, Format, Extra) that make up its header. The other lines in the file represent individual records, each with its timestamp.

### 4.1 Modification and augmentation of timelines

We continued with another modification consisting of extraction additional attributes from existing attributes dependent on the processed dataframe (EVT, FILE). The modifications were same for the desktop and DC files.

In Table 4 we present number of records for each CSV file, number of records after deletion of zero rows and number of records for each data frame – evt and file.

**Table 4:** Number of records for each CSV file.

	The Stolen Szechuan Sauce Case DC	The Stolen Szechuan Sauce Case Desktop	Magnet CTF 2019 Windows Desktop	Magnet CTF 2020 Windows Desktop	Magnet CTF 2022 Windows Laptop	NIST Data Leakage Case
Number of records	1 263 787	1 391 358	2 687 232	9 335 096	13 837 814	3 203 758
Number of records after deletion of zero rows	1 256 180	1 383 481	2 675 531	9 331 454	13 797 332	3 201 948
Number of records – df_evt	86 180	41 721	111 102	130 359	251 666	10 306
Number of records – df_file	843 863	881 454	1 862 397	6 655 042	10 669 920	1 907 945

In both cases (EVT, FILE dataframes) we binarized some of the attributes. Data binarization brings several significant advantages and disadvantages that must be considered when using it. One of the main advantages of binarization is the possibility of aggregating a large amount of data, which simplifies their processing and analysis. On the other hand, one of the disadvantages of binarization is the complexity of finding a suitable aggregation method. Different methods can have different effectiveness, making it difficult to choose the right one for a particular task. In addition, binarization increases the spatial complexity of the data, which can lead to zero columns. These null columns complicate data review and analysis because the analyst may not immediately recognize them. Another problem is that binarization often results in sparse data where there are significantly more zero values than non-zero values. However, binarization allows efficient aggregation, filtering, and joining of the smallest atomic units of data, which is another significant advantage. Some methods, such as formal concept analysis (FCA), even require binary values as input, making binarization necessary for them to function properly.

The second option is to leave the data in its original form, but this approach also has its disadvantages. One of the main limitations is that not all methods can be applied to such untransformed data. Many methods are designed to work optimally with binary or specially modified data, and applying them to raw data can lead to inaccurate or inefficient results. That is why we decided to apply binarization on these data.

### 4.2 Preprocessing of FILE dataframe

For the dataframe **FILE** we continued with another modification for both of timelines consisting of extraction additional attributes.

Firstly, we have modified the records, because the pandas library represents timestamps in nanosecond resolution, the timespan is limited to approximately 584 years. Where the dates were outside the bounds (09/22/1677-04/11/2262), we have changed values to ***NaT*** and then to 01/01/1970 to preserve at least values for time. As we can see in the table below, these changes are only applicable for two datasets (Table 5).

**Table 5:** Number of records in FILE dataframes where date was changed to 01/01/1970.

	The Stolen Szechuan Sauce Case DC	The Stolen Szechuan Sauce Case Desktop	Magnet CTF 2019 Windows Desktop	Magnet CTF 2020 Windows Desktop	Magnet CTF 2022 Windows Laptop	NIST Data Leakage Case
Number of records (df_file) whose dates were changed to 01/01/1970	0	0	0	4 704	0	1488

In the Table 3 we present number of records in FILE dataframes where we changed the dates to 01/01/1970, because they were outside the bounds.

The MACB timestamp attribute had 15 different values, namely: ‘...B', ‘.A.B', ‘M...', ‘MA.B', ‘MA..', ‘M..B', ‘.A..', '‘.. C.', ‘M.C.', ‘MACB', ‘M.CB', ‘MAC.', ‘..CB', ‘.AC.', ‘.ACB'. From the analysis of this attribute, it follows that there is no record in the FILE dataframe that does not have at least one of the time stamps M (modification of file content), A (last access to file), C (modification of MFT item) or B (creation of file) recorded. Since it is a categorical variable, in the next phase it was divided into four separate binary variables M, A, C, B. The values of the Type attribute, which is a description of the MACB attribute, acquired the same values, but were verbally described. For this reason, we omitted the Type attribute during further analysis of the FILE dataframe.

There were four different values that occurred within the Sourcetype attribute, namely: ‘File stat', ‘NTFS file stat', ‘File entry shell item' and ‘NTFS USN change'. This attribute was split into 4 separate binary attributes.

The values of the User attribute were not recorded in the records of the FILE dataframe, for this reason it was also omitted from the further analysis of the data. The same reason applied for the Host and Note attributes. The Version attribute has been omitted due to its immutable value. Since the Short attribute is a shortened version of the Desc attribute, it was omitted from further analysis. The attributes file name (name), file type (filef), folder (directory) or link (link) were extracted from the Desc (description) attribute.

The Filename attribute is also a unique source of data from which we extracted separate attributes regarding the location of the file (dir_appdata, dir_win, dir_user, dir_other) and attributes regarding the file extension - file_executable (files with the extensions exe, msi, cmo and bud), file_graphic (files with the extensions png, jpg, psd and jpeg), file_document (files with the extensions doc, docx, docm, ppt, pptx, pps, ppsx, txt, xls and xlsx), file_ps (files with the extensions ps) and file_other (files with other than previously mentioned extensions).

The Inode attribute was retained as an attribute on which records can be correlated in the next phase of research because it contains unique categorical values.

The Format attribute took six different values, namely: ‘filestat', ‘mft', ‘lnk/shell_items', ‘olecf/olecf_automatic_destinations/lnk/shell_items', ‘winreg/bagmru/shell_items' and usnjrnl'. So it is a categorical variable, which we divided into another 6 binary values.

Similar to Desc, the Extra attribute is a source of unique data that can be a source for other attributes. We extracted from it the attribute is_allocated (is_allocated0, is_allocated1), which speaks about the presence or deleting the file from the disk. Furthermore, the hash of the file (sha_256) and the size of the file (file_size) were extracted as separate attributes.

### 4.3 Preprocessing of EVT dataframe

We also analyzed the **EVT** type records based on the attribute values that were found in the solved case study.

The MACB attribute, as in the case of data from the FILE source, is a categorical attribute that can be decomposed into four binaries. However, there were fewer different values of this attribute than in the case of the FILE source.

The Sourcetype attribute, i.e. a more detailed description of the data source, in this case only acquired the value ‘WinEVTX'. This value is not surprising, since only EVTX records (formerly EVT) appear in the Windows operating system since Vista. For this reason, this attribute was omitted in further analysis.

As in the case of the FILE source, also in this case the type, user, host, version and notes attributes will be omitted during further analysis. Since the format attribute only contains the winevtx value and this information would be duplicated, it was also omitted. We can also omit the inode attribute, since in this case it is the inode of the event source file. The short attribute, as in the case of FILE, is only a shortened version of desc, so we also omitted it.

From the desc attribute, we extracted a new attribute with categorical values that describes the device name (computer_name).

For EVT records, the filename attribute is a categorical variable that contains the file in which the logs are stored. In this case, there were 113 sources, which we sorted into 6 categories, namely Application (filename_application), System (filename_system), Security (filename_security), logs associated with remote access (filename_rdp), logs associated with the use of PowerShell (filename_powershell) and category Other (filename_other).

In the extra attribute, the entire XML string from Windows Event Logs is found. For this reason, it contains unique data from which we have extracted additional attributes. It was the recovered attribute, which takes on the value True if the file has been deleted. Next, there were the attributes user_sid, which identifies the user who created the process, as well as the attribute execution_process_id, which acquires the ID value of the executed process. Attribute task works with the task identifier for the part of the application or component that publishes the event. The event id attribute was also extracted, which we classified into nine groups. We also extracted seven new attributes from the extra attribute, which were related to the values in the keywords field.

## Limitations

None.

## Ethics Statement

The authors have read and follow the ethical requirements for publication in Data in Brief and confirming that the current work does not involve human subjects, animal experiments, or any data collected from social media platforms.

## CRediT authorship contribution statement

**Eva Marková:** Data curation, Writing – original draft. **Pavol Sokol:** Conceptualization, Methodology, Writing – original draft. **Sophia Petra Krišáková:** Visualization, Writing – original draft. **Kristína Kováčová:** Data curation.

## Data Availability

Dataset of Windows operating system forensics artefacts (Original data) (Mendeley Data).
